# Dentists’ Perception of Oral Potentially Malignant Disorders

**DOI:** 10.1016/j.identj.2022.01.004

**Published:** 2022-02-26

**Authors:** Bassel Tarakji

**Affiliations:** Department of Oral and Maxillofacial Surgery and Diagnostic Sciences, College of Dentistry Prince Sattam Bin Abdulaziz University, Al Kharj, Saudi Arabia

**Keywords:** Oral potentially malignant disorder, Clinical feature, Risk factor, Age

## Abstract

**Objectives:**

Dentists play a major role in the diagnosis of oral potentially malignant disorders (OPMDs) that may lead to malignancy. Their knowledge on OPMDs and the risk factors associated with malignant disease needs to be sufficient. The aim of this study was to assess the level of knowledge, attitudes, and awareness of OPMDs amongst general dentists and dental specialists working in Saudi Arabia.

**Material and methods:**

Questionnaires were distributed to dentists working in Saudi Arabia. A total of 303 dentists participated in the study. The questionnaire included 20 questions on knowledge, attitudes, and awareness of OPMDs.

**Results:**

The response rate was 55%. There was no significant difference between general dental practitioners and dental specialists regarding leukoplakia, which is the most common OPMD (*P* > .05) and in identifying tobacco and alcohol as the main risk factors for malignant transformation of OPMDs into cancer (*P* > .05). However, there was a significant difference (*P* < .05) between specialists (75.3%) and general practitioners (52.3%) in the diagnosis of OPMDs. There was a significant difference (*P* < .05) between specialists (63.5%) and general practitioners (28.0%) in recognising the likelihood of malignant transformation of proliferative verrucous leukoplakia. There was a significant difference between specialists (61.2%) and general practitioners (25.2%, *P* < .05) in recognising the erosive form or atrophic type of oral lichen planus, considering that it is more likely to undergo malignant transformation.

**Conclusions:**

Dental specialists have better knowledge and awareness than general dentists regarding OPMDs. Improved continuous education programmes on the risk factors and diagnosis of OPMDs should be organised to train dentists.

## Introduction

Cancer is one of the greatest threats to public health in developed countries.^1^ Squamous cell carcinoma is the most common cancer in the oral cavity. Early detection of oral cancerous and precursor lesions can significantly improve treatment outcomes and prognosis.^2^ New cases of oral cancer are diagnosed each year, with two-thirds of them occurring in Asian countries, including Sri Lanka, Indonesia, India, Pakistan, and Bangladesh.^3^, ^4^, ^5^ Sung et al^1^ indicated to new cases and deaths for 36 cancers and all cancers combined in 2020 in 185 countries. The new cases of lip and oral cavity cancer numbered 377,713 (2.0%). Lip and oral cavity cancers are frequent in South Central Asia and Melanesia. Incidence rates are also high in Eastern and Western Europe and in Australia/New Zealand and have been linked to alcohol consumption, tobacco smoking, human papillomavirus infection for cancers of the oropharyngeal region, and ultraviolet radiation from sunlight exposure for lip cancer.^1^

The detection of oral potentially malignant disorders (OPMDs) in dental clinics is essential to save patients’ lives. Early diagnosis of oral cancer (I and II) can increase survival rates by up to 80%.^6^ Some patients with oral cancer are diagnosed at an advanced stage (stage III and IV) because most patients are asymptomatic in the early stages and do not seek medical help until the development of clear symptoms.^7^ The differential diagnosis and delayed diagnosis of OPMDs that may present without pain affect their prognosis. OPMDs are oral mucosal disorders with an increased risk of malignancy.^8^ Warnakulasuriya et al^9^ indicated that OPMDs include leukoplakia, erythroplakia, proliferative verrucous leukoplakia, oral lichen planus, oral submucous fibrosis, palatal lesions in reverse smokers, lupus erythematous, epidermolysis bullosa, and dyskeratosis congenita. They mentioned in the current update that there is sufficient evidence of an increased risk of oral cancer amongst patients diagnosed with oral lichenoid lesions and amongst those diagnosed with oral manifestation of chronic graft versus host disease.

Odell et al^10^ indicated that dysplasia grade alone should not be used as detrainments of treatment that should be based on overall risk. The risk assessment including: and duration of risk habits, type, age, gender, lesion site, extent and multifocality, clinical features (red, white, speckled, nodular or verrucous), duration suspicious of focal induration, family history to oral cancer. They mentioned that dysplasia grades could not always estimate risk accurately because of sampling error and treatment interventions.

The grade of oral epithelial dysplasia is considered the most significant predictor of malignant transformation and is the best guide for treatment and management.^9^^,^^10^ Tobacco and alcohol use contributes to approximately 75% of all oral cancers.^11^ Various factors are involved in the diagnosis of late or advanced stages of oral cancers and their treatment. Most people are not aware of the signs and symptoms of OPMDs and their correlation with oral cancer; because oral cancer is usually painless and symptom-free in the early stages, patients tend to overlook indicative oral changes.^12^

Previous studies have demonstrated that dentists might be unable to diagnose OPMDs because of the lack of knowledge about the risk factors, signs, and symptoms of squamous cell carcinoma.^12^ Patients regularly visit general dentists, and sufficient knowledge of OPMDs is essential, since these primary dental care providers can detect early lesions and thereby reduce the incidence of oral cancer.^13^ Dentists, who are the first line in detecting early signs of oral cancer, might be unaware of the importance of their role, and some do not include complete head and neck examinations as part of routine examinations.

Although previous studies have evaluated the knowledge that dentists and dental students have about oral cancer,^14^ there are few studies focusing on awareness and knowledge regarding OPMDs. The aim of this study was to evaluate the knowledge, attitudes, and awareness of general dental practitioners and dental specialists regarding OPMDs. In addition, we evaluated the difficulties in diagnosing these lesions.

## Materials and methods

A cross-sectional questionnaire-based survey of general dental practitioners and dental specialists was undertaken in Saudi Arabia.

### Data collection

Google Forms were used to make an electronic copy of the questionnaire. Due to COVID-19 restrictions, online administration of the survey was the best possible option. For this reason, a Google Form was developed and distributed amongst the targeted dentists.

The questionnaire was then distributed through WhatsApp groups and social media to a random sample of dentists working in Saudi Arabia. The author followed a convenience random sampling protocol in recruiting participants. A number of known social media groups and WhatsApp groups of dentists in Saudi Arabia were targeted to invite participation. Most participants in this study represents general and specialist dentists working in Saudi Arabia. To confirm nationwide distribution, the author was keen to target as many as possible dental practitioners with different backgrounds (general dentists and specialists) including holders of DDS/BDS, MSc, PhD, postgraduate diploma at different working fields governmental, and private in the 5 regions of the KSA (Eastern region, Middle region, Northern region, Southern region Western). Furthermore, 5 reminders were sent through WhatsApp groups and other social media platform channels aiming to increase the response rate. Responses to the questionnaire were sensitive to the IP address, assuring no duplicated responses. The purpose of the survey was explained, and dentists were invited to complete the questionnaire. Dentists were assured about the confidentiality and anonymity of the collected data. A timeline of more than 4 months (September 2020 to April 2021) was allowed to collect data, and over this time frequent reminders were sent to dentists to complete the survey items. Informed consent was collected for all participants. The study was approved by the Prince Sattam University Ethical Review Board (REC-HSD-026-2020).

A prevalidated questionnaire was developed based on previous studies.^15^, ^16^, ^17^, ^18^ Before the questionnaire was distributed, a pilot study was performed on a random sample of dentists (n = 30), and the questionnaire was modified according to the feedback obtained. The first part of the anonymous questionnaire consisted of questions related to demographic information, including sex, practice location, practice type, and qualification. The second part included specific questions on OPMDs to assess the knowledge, attitudes, and awareness of general dental practitioners and dental specialists.

### Data analysis

The SPSS statistical package (IBM SPSS Statistics for Windows, version 20.0, released 2011, IBM Corp., Armonk, NY, USA) was used to perform data analysis. Descriptive statistics were calculated for the characteristics of the participating dentists, and frequency tables were generated to illustrate the responses of the dentists to the survey questions. The Chi-square test was performed to assess any possible association amongst questionnaire items, general dental practitioners, and dental specialists. Statistical significance (*P* value) was set at <.05.

## Results

A total of 550 general dental practitioners and dental specialists were contacted. Amongst these, 303 completed the questionnaire (55% response rate), which included 223 male and 80 female participants (Table 1).

**Table 1:** Characteristics of participating dentists (N = 303).

Gender	Female	80 (27%)
	Male	223 (73%)
Practice location	Eastern region	59 (19.5%)
	Middle region	57 (18.8%)
	Northen region	62 (20.5%)
	Southern region	47 (15.5%)
	Western region	78 (25.7%)
Type of practice	Governmental	27 (8.9%)
	Private	267 (88.1%)
	Both	9 (3%)
Qualification	DDS/BDS	218 (71.9%)
	MSc	62 (20.5%)
	PhD	13 (4.3%)
	Postgraduate diploma	10 (3.3%)
Speciality		
	General practice	202 (66.7%)
	Endodontics	16 (5.3%)
	Operative dentistry	21 (6.9%)
	Oral medicine	6 (2%)
	Oral surgery	12 (4%)
	Orthodontics	8 (2.6%)
	Paediatric dentistry	10 (3.3%)
	Periodontics	14 (4.6%)
	Prosthodontics	14 (4.6%)

The response rate for males was 73% and for females was 27%. This study included most of the cites in Saudi Arabia (Table 1). Of the respondents, 218 (71.9 %) were general dental practitioners, 62 (20.5%) had a master's degree; 13 (4.3%) had a PhD, and 10 (3.3%) had a postgraduate diploma. Amongst the dentists, 16 (5.3%) were specialists in endodontics, 202 (66.7%) were general practitioners, 21 (6.9%) were operative dentists, 6 (2%) were oral medicine doctors, 12 (4%) were oral surgeons, 8 (2.6%) were specialists in orthodontics, 10 (3.3%) were pediatric dentists, 14 (4.6%) were specialists in periodontics, and 14 (4.6%) were specialists in prosthodontics (Table 1). A total of 128 (42.2%) dentists detected fewer than 10 cases of OPMDs per year and 158 (51.1%) dentists detected more than 10 cases per year (Table 2). A limited number of dentists (5.6%) did not detect any case. This indicates that OPMDs are detectable by dentists in Saudi Arabia. A limited number of dentists (22 [7.3%]) were interested in attending oral medicine as a favourite course (Table 2).

**Table 2:** Knowledge, attitudes, and awareness of participating dentists about oral potentially malignant disorders (N = 303).

**How many cases of potentially malignant disorders do you detect per year?** I do detect any one, 17 (5.6%) Fewer than 10 cases, 128 (42.2%) More than 10 cases, 158 (51.1%)
**What is your favourite dental course to attend?** Aesthetic dentistry and others, 198 (65.3%) Oral medicine, 22 (7.3%) Not interested, 83 (27.4%)
**How can skills of dental practitioners in the diagnosis of oral potentially malignant disorders be improved?** Continuous clinical courses after graduation, 160 (52.8%) Increase teaching hours in undergraduate programmes in oral medicine, 84 (27.7%) I do not know, 59 (19.5%)
**Leukoplakia is the most common type of oral potentially malignant disorder** Agree, 221(72.9%) Disagree, 35 (11.6%) Not sure, 47 (15.5%)
**Erythroplakia is more likely to show malignant transformation** Agree, 173 (57.1%) Disagree, 41 (13.5%) Not sure, 89 (29.4%)
**Tobacco, alcohol, and cigarettes are the main risk factors for transforming oral potentially malignant disorders to cancer** Agree, 167 (55.1%) Disagree, 39 (12.9%) Not sure, 97 (32%)
**Age older than 40 years old carries more potential for oral potentially malignant disorders to become malignant** Agree, 137 (45.2%) Disagree, 26 (8.6%) Not sure, 140 (46.2%)
**Oral potentially malignant disorders on the lateral surface of the tongue are more likely to show malignant transformation** Agree, 113 (37.3%) Disagree, 31 (10.2%) Not sure, 159 (52.5%)
**Proliferative verrucous leukoplakia is more likely to show malignant transformation** Agree, 115 (38%) Disagree, 26 (8.2%) Not sure, 162 (53.5%)
**An erosive form or atrophic type of oral lichen planus is more likely to show malignant transformation** Agree, 107 (35.5%) Disagree, 34 (11.2%) Not sure, 162 (53.5%)
**A dentist should wait 3 weeks before taking a biopsy of the abnormal lesion in the mouth** Agree, 125 (41.3%) Disagree, 30 (9.9%) Not sure, 148 (48.8%)
**Do you experience difficulties in identifying oral potentially malignant disorders on clinical examination?** Yes, 178 (58.7%) No, 32 (10.6%) Not sure, 93 (30.7%)
**When you recognise abnormal oral potentially malignant disorders, do you refer the patient to a specialist in oral surgery?** Yes, 170 (56.1%) No, 28 (9.2%) Not sure, 105 (34.7%)
**When you recognise abnormal oral potentially malignant disorders, do you refer the patient to a specialist in oral medicine?** Yes, 127 (41.9%) No, 52 (40.9%) Not sure, 124 (34.7%)
**The confirmation diagnosis of oral potentially malignant disorders depending on histological examination when recognise abnormal oral lesion** Yes, 147 (48.5%) No, 17 (5.6%) Not sure, 139 (45.9%)
**Do you think that a specialist in oral medicine or oral pathology is the typical specialist to detect oral potentially malignant disorders?** Yes, 169 (55.8%) No, 19 (6.3%) Not sure, 115 (38%)

There were 160 (52.8%) dentists who preferred continuous clinical courses after graduation to improve skills in the diagnosis of OPMDs; 84 (27.7%) dentists recommended increasing teaching hours at the undergraduate level of oral medicine to improve skills in the diagnosis of OPMDs (Table 2). Of the participants, 221 (72.9%) believed that leukoplakia is the most common type of precancerous lesion and 173 (57.1%) indicated that erythroplakia is more likely to show malignant transformation.

There were 167 (55.1%) participants who mentioned that tobacco, alcohol, and cigarettes are the main risk factors for the malignant transformation of OPMDs into cancer (Table 2). Of the respondents, 137 (45.2%) attended to patients aged 40 years or older for the diagnosis of OPMDs (Table 2); 113 (37.3%) paid attention to the signs of OPMDs on the lateral surface of the tongue, which are more likely to become malignant (Table 2). A low number of participants (115 [38%]) recorded that proliferative verrucous leukoplakia has a greater potential for malignant transformation than other types of leukoplakia (Table 2). This study showed that 107 (35.5%) respondents believed that an erosive or atrophic type of oral lichen planus (OLP) is more likely to show malignant transformation (Table 2). There were 125 (41.3%) participants who indicated that they would wait 3 weeks before taking a biopsy of the abnormal lesion in the mouth (Table 2). There were 178 (58.7%) respondents who faced difficulties in the clinical diagnosis of OPMDs; 170 (56.1%) participants preferred to refer their patients to oral surgery clinics for the detection of OPMDs and 127 (41.9%) referred their patients to oral medicine clinics (Table 2). A total of 147 (48.5%) patients reported a diagnosis of OPMDs based on histological examination when recognising abnormal oral lesions (Table 2); 169 (55.8%) respondents believed that specialists in oral medicine or oral pathology were the appropriate specialists for detecting OPMDs (Table 2).

## Discussion

This study reflects the knowledge, attitudes, and awareness of dental practitioners, which included general practitioners and specialists in dentistry, regarding the clinical presentation of OPMDs, predisposing factors, and difficulties in the diagnosis of OPMDs in general dental practice. There are few published studies regarding the evaluation of knowledge, awareness, and opinions of dentists on OPMDs compared to oral cancer.^19^, ^20^, ^21^ Similar response rates have been reported in other studies on the same topic conducted amongst dentists,^19^, ^20^, ^21^, ^22^, ^23^ but the current response rate is higher than 40% as reported by Alonge and Narendran.^24^

This study showed that there was no significant difference (*P* > .05) between general dental practitioners and specialists regarding the recognition of leukoplakia as the most common type of OPMD (Table 3). Hassona et al^18^ reported that leukoplakia was the best-known OPMD and was identified in 45% of participants; moreover, amongst the general dental practitioners and dental specialists, 74.8% and 68.2% identified leukoplakia as the most common OPMD, respectively. In addition, there was no significant difference between them (*P* > .05) regarding the recognition of tobacco, alcohol, and cigarettes as the main risk factors for malignant transformation of OPMDs into cancer (Table 3). The response rate regarding the recognition of tobacco, alcohol, and cigarettes as the main risk factors was very similar to those reported in other studies.^25^,^26^

**Table 3:** Association between survey items and type of qualification (ie, general dental practitioners vs specialist dentists).

Statement/question about oral potentially malignant disorders	% of “agree/yes” answers based on qualification		
	Qualification		
	GP	Specialist	*P* value
Leukoplakia is the most common type of oral potentially malignant disorder	74.8	68.2	.50
Tobacco, alcohol, and cigarettes are the main risk factors for transforming oral potentially malignant disorders to cancer	54.1	57.6	.82
Oral potentially malignant disorders on the lateral surface of the tongue are more likely to show malignant transformation	31.7	51.8	.00*
Proliferative verrucous leukoplakia is more likely to show malignant transformation	28	63.5	.00*
An erosive form or atrophic type of oral lichen planus is more likely to show malignant transformation	25.2	61.2	.00*
Do you experience difficulties in identifying oral potentially malignant disorders on clinical examination?	52.3	75.3	.00*
The confirmation diagnosis of oral potentially malignant disorders depending on histological examination when recognise abnormal oral lesion	40.8	68.2	.00*

⁎ Denotes significant difference at *P* < .05 as indicated by Chi-square statistics.

This study showed a significant difference (*P* < .05) between dental specialists (75.3%) and general dental practitioners (52.3%) regarding difficulties in detecting OPMDs (Table 3).

The diagnosis of OPMDs depends on adequate clinical skills and histological investigation. I believe that general dentists might focus just on the dentition and the immediately surrounding periodontal tissue. When examining the oral cavity, general practitioners may emphasise teeth more than oral tissues. General dentists may have difficulties in the detection and differential diagnosis of a wide spectrum of OPMDs due to fewer clinical diagnostic skills compared with specialists. Specialists typically have better education and knowledge than general practitioners; therefore, they understand the difficulties in the diagnosis of such lesions. Chiang et al^27^ reported that the specialist diagnoses exhibited higher specificity, positive predictive value, and accuracy than those of primary examiners (general dental clinicians). Leuci et al^28^ reported that most general dental practitioners refer their patients to specialists; therefore, they might not be involved in the diagnosis of OPMDs.

In addition, this study showed that there was a significant difference (*P* < .05) between general practitioners (31.7%) and specialists (51.8%) in recognising that OPMDs on the lateral surface of the tongue might undergo malignant transformation (Table 3). Bsoul et al^29^ reported that more than 30% of all head and neck cancer lesions occur on the tongue, followed by floor of the mouth lesisons. Kujan et al^30^ indicated that more dental students in their sixth year recognise the tongue as the most likely location of oral cancer development than dental students in their fourth or fifth year. Al-Maweri et al^31^ reported that 85% of students identified tongue as the most likely location for the malignant transformation to oral cancer, especially the students in an advanced academic year.

This study showed that there was a significant difference (*P* < .05) between dental specialists (63.5%) and general dental practitioners (28.0%) in the recognition of the likelihood of malignant transformation of proliferative verrucous leukoplakia (Table 3). Parashar^32^ reported that proliferative verrucous leukoplakia is a multifocal form of progressive leukoplakia with a high rate of malignant transformation that requires early recognition by oral health care providers for proper management. Dental specialists in this study showed better knowledge than general practitioners regarding the possibility of malignant transformation of proliferative verrucous leukoplakia. However, the participants in other studies, which included general dentists and specialists, showed better knowledge than dentists.^33^ Wimardhani^33^ reported that 46 out of 404 participants (11.4%) identified leukoplakia or erythroplakia as conditions likely to undergo malignant transformation. This indicates that dentists in Saudi Arabia are well educated and knowledgeable.

This study showed that there was a significant difference between dental specialists (61.2%) and general dental practitioners (25.2%, *P* < .05) in recognising the erosive or atrophic type of OLP, which is more likely to show malignant transformation (Table 3). Another study^20^ reported that 22.6% of participants identified OLP as a type of OPMD that can undergo malignant transformation. Abati et al^3^ reported that erosive and atrophic OLP can cause severe pain and interfere with speech and swallowing. Erosive OLP carries the highest risk of malignant transformation, followed by atrophic OLP.^20^

This study showed that there was a significant difference between dental specialists (68.2%) and general practitioners (40.8%, *P* < .05) regarding the diagnosis of OPMDs based on histological examination when recognising abnormal oral lesions (Table 3). These results are similar to those reported previously,^21^ which reported that 70% of the dentists with more experience were aware of a definitive diagnosis depending on the histological features of OPMDs. It is suggested that the presence of dysplasia is considered the most useful indicator of possible malignant transformation.^3^

Based on the results of this study, it seems that there is a varying level of knowledge and awareness amongst the participants that lesions such as leukoplakia, erythroplakia, and OLP are the most common OPMDs.

One possible strength of this study is the comparison of the level of knowledge, attitudes, and awareness between general dental practitioners and dental specialists regarding OPMDs. Continuing education courses can have a positive impact on their knowledge and practices.^6^ Primary oral health care providers play a vital role in screening patients for OPMDs, and OPMDs must be a regular part of continuing dental education courses.

## Limitations

The limitation of this study is the small sample size. There could be a bias in selection of dentists, in that motivated dentists could have responded and not dentists who lack interest. Also, using a convenience sampling method can be amongst the limitations of the study. However, involvement of practicing dentists from the various geographic locations of Saudi Arabia, male and female dentists, dentists practicing in the private and public sectors, general dentists and specialists can add to the power and generalisability of the findings.

## Conclusions

Dentists’ knowledge, attitudes, and awareness regarding OPMDs are limited in some respects. In general, dental specialists have better knowledge and awareness than general dental practitioners regarding OPMDs. Therefore, improvement in education courses and training for both general dental dentists and dental specialists are crucial for improving early diagnosis of OPMDs, and high priority should be given to the education on the prevention of malignant transformation. More studies in this field are highly recommended for motivating dentists to obtain further training in the early detection of OPMDs in clinical practice.

## Appendix Supplementary materials

Image, application 1
