# Supplementary material for: Dentists’ Perception of Oral Potentially Malignant Disorders
Source: Int Dent J. 2022 Feb 26;72(3):414–9. doi: 10.1016/j.identj.2022.01.004 (PMC9275301; doi:10.1016/j.identj.2022.01.004)
Supplement: Supplementary file 1 [file mmc1.doc]

Dentists’ perception of oral potentially malignant disorders

Please complete the following information and tick/circle as appropriate

| ***Section 1*** | | | | | | | |
| --- | --- | --- | --- | --- | --- | --- | --- |
| **Gender** | *Male* | | |  |  | | |
|  | *Female* | | |  |  | | |
|  | | | | | | | |
| **Type of Practice** | *Governmental* | | |  |  | | |
|  | *Private* | | |  |  | | |
|  | *Both* | | |  |  | | |
|  | | | | | | | |
| **Practice location** | | | *Eastern* region | | |  | |
|  |  |  | *Middle region* | | |  | |
|  |  |  | *Northen region* | | |  | |
|  |  |  | Southern *region* | | |  | |
|  |  |  | Western region | | |  | |
|  |  |  |  |  |  |  |  |
|  |  |  |  |  |  |  |  |
|  |  |  |  |  |  |  |  |
|  |  |  |  |  |  |  |  |
|  |  |  |  |  |  |  |  |
|  |  |  |  |  |  |  |  |
|  |  |  |  |  |  |  |  |
|  |  |  |  |  |  |  |  |
| **Qualification** | | *DDS/BDS* | | | | |  |
|  |  | *MSc* | | | | |  |
|  |  | *PhD* | | | | |  |
|  |  | *Post graduate Diploma* | | | | |  |
|  |  |  |  |  |  |  |  |
|  | | | | | | | |
| **Specialty** | | *General practice* | | | | |  |
|  |  | *Endodontics* | | | | |  |
|  |  | Operative Dentistry | | | | |  |
|  |  | Oral Medicine | | | | |  |
|  |  | Oral Surgery | | | | |  |
|  |  | Orthodontics | | | | |  |
|  |  | Paediatric Dentistry | | | | |  |
|  |  | Periodontics | | | | |  |
|  |  | Prosthodontics | | | | |  |
|  |  |  |  |  |  |  |  |

**Section 2:**

**Knowledge, attitudes and awareness of participating dentists about oral potentially malignant disorders**

1) How many cases of potential malignant disorders do you detect per year?

a) I do detect any one

b) less than 10

c) More than 10

2) What is your favorite dental course to attend

1. Esthetic dentistry and others
2. Oral medicine
3. Not interested

3) How can improve skills of general practitioners in diagnosis of potential malignant disorders

1. Continuous clinical courses after graduation
2. Increase teaching hours at undergraduate in oral medicine
3. I do not know

4) Leukoplakia is the most common type of oral potentially malignant disorders

1. Agree
2. Disagree
3. Not sure

5) Erythroplakia is more likely to show malignant transformation?

1. Agree
2. Disagree
3. Not sure

6) Tobacco, Alcohol and cigarettes are the main risk factor of transforming oral potentially malignant disorders to cancer?

1. Agree
2. Disagree
3. Not sure

7) Age above 40 years old are more potential for oral potentially malignant disorders to become malignant?

1. Agree
2. Disagree
3. Not sure

8) Oral potentially malignant disorders on lateral surface of the tongue is more likely of to show malignant transformation?

1. Agree
2. Disagree
3. Not sure

9) Proliferative verrucous leukoplakia is more likely to show malignant transformation

1. Agree
2. Disagree
3. Not sure

10- A erosive form or atrophic type of oral lichen planus is more likely to show malignant transformation?

1. Agree
2. Disagree
3. Not sure

11- Dentist should wait for three weeks before taken biopsy of the abnormal

lesion in the mouth

1. Agree
2. Disagree
3. Not sure

12- Do you find difficulties in identifying oral potentially malignant disorders from clinical examination?

1. Yes
2. No
3. Not sure

13) when recognize abnormal oral potentially malignant disorders? Do you refer patient to specialist in oral surgery?

1. Yes
2. No
3. Not sure

14) when recognize abnormal oral potentially malignant disorders? Do you refer patient to specialist in oral medicine?

1. Yes
2. No
3. Not sure

15) The confirmation diagnosis of oral potentially malignant disorders depending on histological examination when recognize abnormal oral lesion

1. Yes
2. No
3. Not sure

16) Do you think that specialist in oral medicine or oral pathology are the typical specialist to detect oral potentially malignant disorders?

1. Yes
2. No
3. Not sure
